# Gastric Electrical Pacing Reduces Apoptosis of Interstitial Cells of Cajal via Antioxidative Stress Effect Attributing to Phenotypic Polarization of M2 Macrophages in Diabetic Rats

**DOI:** 10.1155/2021/1298657

**Published:** 2021-02-28

**Authors:** Hongcai Wang, Kaile Zhao, Ying Ba, Tao Gao, Ning Shi, Qiong Niu, Chengxia Liu, Yan Chen

**Affiliations:** ^1^Department of Neurology, Binzhou Medical University Hospital, Binzhou, Shandong, China; ^2^Department of Gastroenterology, Binzhou Medical University Hospital, Binzhou, Shandong, China

## Abstract

**Background:**

Gastric electrical pacing (GEP) could restore interstitial cells of Cajal in diabetic rats. M2 macrophages contribute to the repair of interstitial cells of Cajal injury though secreting heme oxygenase-1 (HO-1). The aim of the study is to investigate the effects and mechanisms of gastric electrical pacing on M2 macrophages in diabetic models.

**Methods:**

Sixty male Sprague-Dawley rats were randomized into control, diabetic (DM), diabetic with the sham GEP (DM+SGEP), diabetic with GEP1 (5.5 cpm, 100 ms, 4 mA) (DM+GEP1), diabetic with GEP2 (5.5 cpm, 300 ms, 4 mA) (DM+GEP2), and diabetic with GEP3 (5.5 cpm, 550 ms, 4 mA) (DM+GEP3) groups. The apoptosis of interstitial cells of Cajal and the expression of macrophages were detected by immunofluorescence technique. The expression levels of the Nrf2/HO-1 and NF-*κ*B pathway were evaluated using western blot analysis or immunohistochemical method. Malonaldehyde, superoxide dismutase, and reactive oxygen species were tested to reflect the level of oxidative stress.

**Results:**

Apoptosis of interstitial cells of Cajal was increased in the DM group but significantly decreased in the DM+GEP groups. The total number of macrophages was almost the same in each group. In the DM group, M1 macrophages were increased and M2 macrophages were decreased. However, M2 macrophages were dramatically increased and M1 macrophages were reduced in the DM+GEP groups. Gastric electrical pacing improved the Nrf2/HO-1 pathway and downregulated the phosphorylation of NF-*κ*B. In the DM group, the levels of malonaldehyde and reactive oxygen species were elevated and superoxide dismutase was lowered, while gastric electrical pacing reduced the levels of malonaldehyde and reactive oxygen species and improved superoxide dismutase.

**Conclusion:**

Gastric electrical pacing reduces apoptosis of interstitial cells of Cajal though promoting M2 macrophages polarization to play an antioxidative stress effect in diabetic rats, which associates with the activated Nrf2/HO-1 pathway and the phosphorylation of NF-*κ*B pathway.

## 1. Introduction

For centuries, gastrointestinal motility disorders, one of the most frequently occurring diseases, have kept perplexed people's lives with decades of diabetes mellitus [1]. Gastroparesis, namely delayed gastric emptying, is a disorder that slows or reduces the food transit from the stomach to the small intestine without mechanical obstruction. However, effective therapies for gastroparesis remain elusive with limited roles and side effects. Fortunately, nondrug treatments, such as electroacupuncture (EA) and gastric electrical stimulation (GES), are gradually taken seriously because of its apparent efficacy without side effects. In particular, long pulse GES, for it induces slow waves also referred to as gastric electrical pacing (GEP), has a direct impact on gastric motility. At present, GEP develops as an alternative therapy of gastroparesis, but the mechanisms underlying its efficacy remain unclear.

Interstitial cells of Cajal (ICCs) serve as pacemakers and generate slow waves spontaneously in the stomach. Defect of ICCs has been consistently found in both humans and animal models with diabetic gastroparesis [2, 3]. We have reported that long-pulse GES could repair the injured ICCs partly by IGF-1 signaling pathway and enhancing the proliferation of ICCs [4, 5]. However, apoptosis of ICCs was also certificated in the stomach of gastroparesis [6]; the effects and mechanisms of GEP on apoptosis of ICCs need to be further clarified.

Macrophages have two different phenotypes: proinflammatory M1 macrophages and anti-inflammatory M2 macrophages. They can be transformed into each other in a certain internal environment. Studies have shown that gastrointestinal motility disorder did not occur in diabetic mice with the absence of macrophages, suggesting that macrophages may participate in the development of gastroparesis [7]. It is also reported that phenotypic polarization of M2 macrophages could improve the delayed gastric emptying [8]. Further studies have shown that there was no significant change in the total number of macrophages in animal models and patients of diabetic gastroparesis, while CD206+ M2 macrophages were selectively decreased, accompanied by ICC deletion, resulting in delayed gastric emptying [9–11]. Accordingly, GEP may promote the phenotypic polarization of M2 macrophages to improve ICC expression and gastric emptying.

Heme oxygenase-1 (HO-1) is a widely existing antioxidant defense enzyme, which is not expressed or low expressed in normal tissues, but upregulated during stress playing an anti-inflammatory, antioxidative, and antiapoptotic role. In the gastrointestinal tract, HO-1 is mainly produced and expressed by resident M2 macrophages [12]. Recently, in a study of diabetic gastroparesis model, it has been found that the downregulation of HO-1 could not resist oxidative stress injury, which leads to the destruction of ICCs network and delayed gastric emptying, but upregulation of HO-1 could repair the injury of ICCs and improve the delayed gastric emptying [13, 14]. Similarly, Tian LG et al. reported that the expression of HO-1 in gastric antrum of diabetic gastroparesis mice was significantly decreased, and EA could improve the expression of HO-1 and gastric motility [15]. Therefore, we speculate that GEP may play a key role in improving gastric motility disorder by upregulating HO-1 to repair ICC injury.

Also, studies have shown that HO-1 can protect and reverse oxidative stress damage to ICCs [13]. Stem cell factor (also known as SCF, c-kit ligand) is essential for survival and maintenance of ICCs, but there are few reports about the correlation between HO-1 and SCF. Recent studies [16] have shown that the NF-*κ*B signaling pathway was activated in diabetic gastrointestinal motility disorders and the decrease expression of SCF/c-kit causes the increase of ICC apoptosis, suggesting that the activation of NF-*κ*B signaling may be an important factor in ICC apoptosis. It is also reported that nuclear factor erythroid 2-related factor 2 (Nrf2) plays a critical role in defending against inflammation in different tissues via activation of phase II enzyme HO-1 and inhibition of the NF-*κ*B signaling pathway [17]. Other studies [18] have shown that HO-1 can inhibit the phosphorylation of NF-*κ*B p65, promote the binding of anti-apoptotic genes to NF-*κ*B, and promote gene transcription to play an antiapoptotic role. In addition, studies in human lung fibroblasts have shown that nuclear transcription factor NF-*κ*B binds to enhancers of SCF gene and promotes SCF transcription [19]. We assumed that HO-1 may play an antiapoptotic role by inhibiting the phosphorylation of NF-*κ*B p65.

In this study, we aimed to explore the effects of GEP on ICC apoptosis and phenotypic polarization of macrophages and to investigate the possible mechanisms of renovation of ICC injury in diabetic rats.

## 2. Materials and Methods

### 2.1. Animals

**Figure 1:**
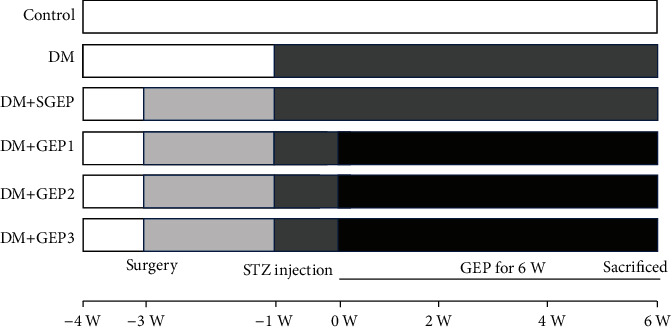
Sketch map of the experimental procedure. The six columns represent different groups as control (normal control group), DM (diabetes group), DM+SGEP (diabetic+sham GEP group), DM+GEP1 (diabetic+GEP1 group), DM+GEP2 (diabetic+GEP2 group), and DM+GEP3 (diabetic+GEP3 group). The whole experimental process maintained for 10 weeks. All rats were adapted to the laboratory conditions for one week (from -4 W to -3 W). All electrodes were implanted by the same surgeon in the serosa of the greater curvature of rats in the DM+SGEP and DM+GEP groups (-3 W). Two weeks later (-1 W), streptozotocin (STZ) was injected to establish diabetic models. Gastric electric pacing (GEP) was carried out in the DM+GEP group 30 min/day for 6 weeks.

Male Sprague-Dawley rats (weighing 160-200 g, *N* = 60) were purchased from Jinan Pengyue Experimental Animal Breeding Co. Ltd. (Shandong, China) and were kept in the suitable laboratory conditions (22-23°C, 12/12 h light-dark cycle) with food and water *ad libitum*. The experimental procedures were implemented, following the ethical guidelines from the Animal Care and Use Committee of Binzhou Medical University Hospital Laboratory Animal Ethical Committee. The rats were randomly divided into the normal control, diabetes (DM), diabetic+sham GEP (DM+SGEP), and diabetic+GEP (DM+GEP) groups (Figure 1).

### 2.2. Surgical Procedures

After an overnight fast, the rat was anesthetized with sodium pentobarbital (37.5 mg/kg, ip). To implant stimulation electrodes, a 2 cm ventral midline incision was made and the stomach was exteriorized. A pair of electrodes was imbedded 2 cm above the pylorus in the serosa of the greater curvature. The ends of the electrode wires were inserted through the abdominal wall, tunneled subcutaneously out of the back neck, and fixed carefully. When doing stimulation, the wires were connected to a programmed electrical stimulator (YC-2, Chengdu Instrument Factory, China).

### 2.3. Diabetic Model

After 2 weeks of recovery from the surgery, diabetic models were established using streptozotocin (STZ, 60 mg/kg, ip, Alexis Biochemical, United States). Animals in the control group were given equal volume of solvent. After one week, blood glucose of the tail vein was measured and diabetes was confirmed twice with a blood glucose > 16.7 mmol/L.

### 2.4. Experimental Protocol

According to the parameters used in our previous study which were all effective in ICC alteration [4, 5], the DM+GEP group was further divided into three subgroups (each *n* = 10): DM+GEP1 (5.5 cpm, 100 ms, 4 mA), DM+GEP2 (5.5 cpm, 300 ms, 4 mA), and DM+GEP3 (5.5 cpm, 550 ms, 4 mA). The rats in the DM+GEP groups were treated with gastric electrical stimulation 30 min daily starting at 9 : 00 am, while the rats in the DM + SGEP group were treated with sham gastric electrical stimulation (stimulation electrodes connected to the stimulator without electric current). To avoid the influence of stress, the rats were put into a quiet and dark place during the stimulation. After stimulation for a period of 6 weeks, all rats were sacrificed to obtain gastric antrum tissues for western blot and immunostaining.

### 2.5. Immunostaining

The whole mount staining was used to test the apoptosis of ICCs. The tissue was blocked by normal rabbit serum and then the primary antibody c-kit (1 : 100, Invitrogen, USA) at 4°C overnight. The apoptosis of ICCs was detected with TUNEL Kit (labeling solution : enzyme solution = 9 : 1, Roche, Germany) for 1 h. After incubation with fluorescent labeled secondary antibodies, DAPI was used to label the nucleus. Then, the apoptotic ICCs could be observed under a fluorescence microscope.

For the section staining, the tissue of gastric antrum was fixed in 4% paraformaldehyde for 24 h and embedded into paraffin blocks and sliced about 6 *μ*m. The sections were gradually deparaffinized in xylene and hydrated in a graded ethanol solution. After inhibiting the endogenous peroxidase activity by adding 0.3% hydrogen peroxide, the sections were microwaved (750 W) to expose antigens. Through nonspecific reaction blocked by normal rabbit serum, the primary antibodies NF-*κ*B (1 : 100, Proteintech, USA), CD68 (1 : 100, Abcam, USA), CD86 (1 : 100, Novus, USA), CD206 (1 : 100, Proteintech, USA), and HO-1 (1 : 100, Proteintech, USA) were added to reveal different proteins.

With incubation with horseradish peroxidase-linked streptavidin marked secondary antibody for 1 h at room temperature, some sections were used to test the expression of NF-*κ*B immunohistochemistry technology. And then a fresh 3,3′-diaminobenzidine solution was used to reflect the positive protein. After being counterstained and dehydrated, glass cover slips are mounted onto the slides.

To test immunoreactivity of CD68, CD86, CD206, and HO-1, fluorescent-labeled secondary antibodies were used for 1 h at room temperature. DAPI was added to stain the nucleus. All of sections were sealed with the antifluorescence quenching agent and observed under a fluorescence microscope.

### 2.6. Western Blot

Fresh-frozen distal stomach samples were grinded into cell suspension on ice using a homogenizer and homogenized in RIPA buffer with a protease inhibitor. After centrifugation at 12000 rpm for 5 min in 4°C, the supernatants were taken as the total protein. Loading buffer was mixed with the protein to scale prior to sodium dodecyl sulfate polyacrylamide gel electrophoresis. The total protein was separated by 10% SDS-PAGE and then transferred to a PVDF membrane. According to molecular weight, these membranes were incubated with primary antibodies against c-kit (1 : 200, Invitrogen, USA), SCF (1 : 1000, R&D Systems, USA), NF-*κ*B (1 : 2000, Proteintech, USA), p-NF-*κ*B (1 : 1000, CST, Germany), IKK (1 : 600, Proteintech, USA), HO-1 (1 : 1000, Proteintech, USA), Nrf2 (1 : 5000, Proteintech, USA), and GAPDH (1 : 1000, Beyotime, China) at 4°C overnight. Then, secondary antibody was added for 2 h at 37°C. Signal detection was visualized using an enhanced chemiluminescent agent, and the blot was present and detected by autoradiography.

### 2.7. Measurement of Malonaldehyde (MDA) Activity

MDA, a marker of oxidative stress, was determined by thiobarbituric acid method following the kit instruction (Nanjing Jiancheng Bioengineering Institute, China). Briefly, tissue was homogenized in Tris-HCl buffer at the ratio of 1 : 10 (mg/mL) under ice bath conditions. The supernatant was taken to be measured after centrifugation at 2500 rpm for 10 min. 2.5 mL thiobarbituric acid was added to the supernatant, and then, the solution was incubated at 95°C for 40 min. After that, samples were cooled with the flow water and centrifuged at 4000 rpm for 10 min. The MDA concentration (nmol/g) was measured spectrophotometrically at 532 nm.

### 2.8. Measurement of Superoxide Dismutase (SOD) Activity

SOD is a key enzyme in the dismutation of superoxide radicals, providing cellular defense against reactive oxygen species. SOD activity was ultimately determined using assay kits (Nanjing Jiancheng Bioengineering Institute, China). Distal stomach samples were homogenized in Tris-HCl buffer (1 : 10) under ice bath conditions and then centrifuged at 2500 rpm for 10 min. The supernatant was collected and mixed with working solutions, incubating at 37°C for 20 min. The absorbance of each well was determined by enzyme-labeled instrument at 450 nm. The level of inhibition was measured, and the SOD level in U/g was thus determined.

### 2.9. Detection of Reactive Oxygen Species (ROS)

Levels of ROS, a number of reactive molecules and free radicals derived from molecular oxygen, were detected by labeling with the oxidant-sensitive probe 2,7-dichlorofluorescein diacetate (DCF-DA) according to the instruction of Reactive Oxygen Species Assay Kit (Beyotime, China). After fresh tissues were homogenized and centrifuged at 1500 rpm for 5 min at 4°C, the supernatants were collected for the ROS assay. Briefly, cells were incubated with DCFH-DA at a final concentration of 10 *μ*M for 20 min at 37°C. After removal of free DCFH-DA, the ROS levels were analyzed immediately with a flow cytometry (CytoFLEX, Beckman Coulter, USA).

### 2.10. Statistical Analysis

All values were expressed as the means ± SEM, and one-way ANOVA was adopted to analyze the difference among multiple groups. *P* < 0.05 was adopted as a statistically significant difference. All statistical analyses were done using SPSS 24.0 (SPSS Inc., Chicago, IL).

## 3. Results

### 3.1. Effects of GEP on Apoptosis of ICCs and SCF/c-Kit Pathway

**Figure 2:**
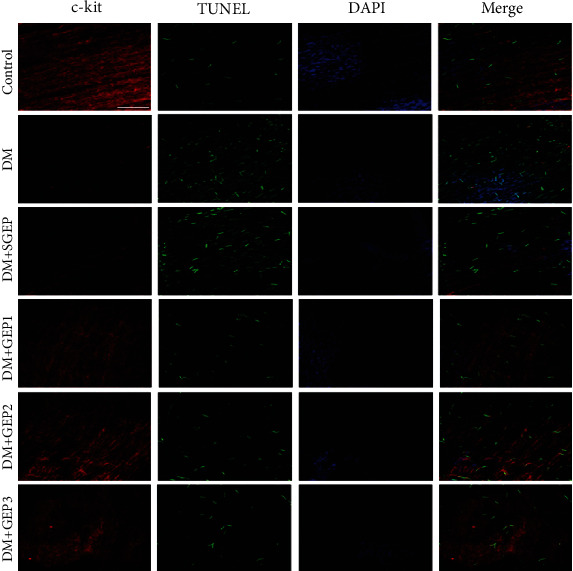
The alteration of the apoptosis of interstitial cells of Cajal (ICCs). The c-kit-positive ICC expression changes (staining with red) were investigated in different groups. The number of TUNEL-positive ICCs (staining with green) was counted to exhibit apoptotic cells (*n* = 6 in each group). Scale bar = 100 *μ*m.

The apoptosis of ICCs in each group is shown in Figure 2. In the control group, a lot of ICCs with few TUNEL+ cells were observed in the antrum. However, a lot of TUNEL+ cells were seen in the DM group, with significantly reduced c-kit+ cells. There was no significant difference of apoptotic ICCs between the DM+SGEP group and the DM group. From Figure 1, we could see a lot of ICCs with rare TUNEL+ cells distributed in the antrum of stomach in the DM+GEP groups.

**Figure 3:**
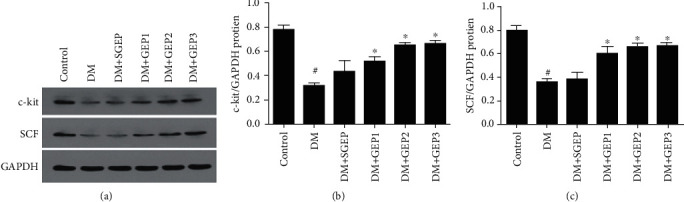
Expressions of c-kit and stem cell factor (SCF) protein in gastric antrum sample of different groups. (a) The altered expression of c-kit and SCF protein was tested in different groups by western blot analysis. GAPDH antibody was used as a loading control. (b, c) The relative grey ratio of protein bands was statistically evaluated in each group (*n* = 6 in each group). Data depicted as the mean ± SEM. ^#^Compared with the control group, *P* < 0.05. ^∗^Compared with the DM group, *P* < 0.05.

Figure 3 demonstrates the expression of c-kit and SCF protein. In the DM group, the c-kit expression was significantly decreased compared with the control group (*P* < 0.001). The expression of c-kit in the DM+SGEP group was not significantly altered compared to that in the DM group (*P* = 0.504). However, the c-kit expression was dramatically improved in the DM+GEP groups compared with the DM group (*P* = 0.042, *P* < 0.001, and *P* < 0.001, respectively). For the expression of SCF protein, it was obviously decreased in the DM group compared with the control group (*P* < 0.001). There was no significantly difference in the DM+SGEP group compared to the DM group (*P* = 1.000). Nevertheless, the SCF expression was obviously increased in the DM+GEP groups compared with the DM group (*P* = 0.080, *P* < 0.001, and *P* < 0.001, respectively).

### 3.2. Effects of GEP on Macrophages

**Figure 4:**
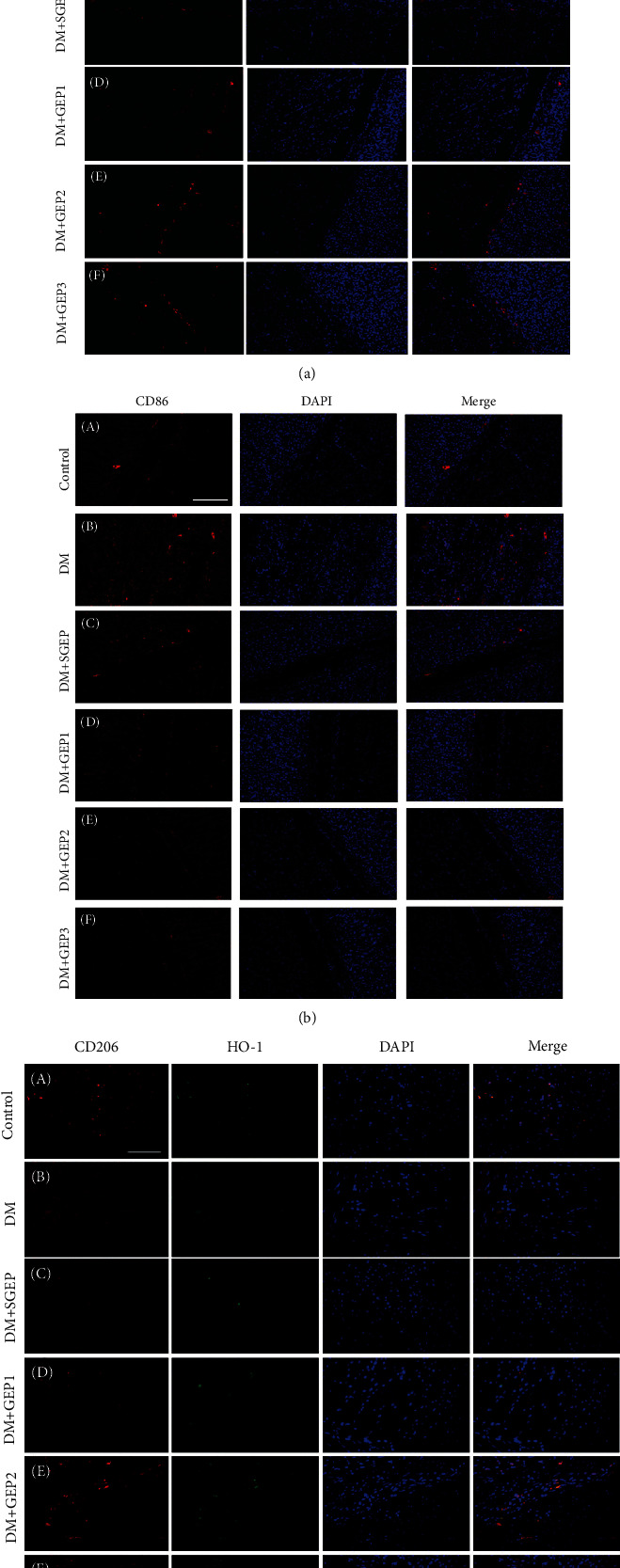
Differential effects of GEP stimulation on the polarization profiles of M1 and M2 macrophages. (a) The expression of CD68 positive macrophages was explored in the gastric antrum tissue of rats. (b) The expression of M1 macrophages (CD86 positive) was observed in different group. (c) The CD206- (red) and HO-1- (green) positive cells represent the expression of M2 macrophages (*n* = 6 in each group). All scale bars = 100 *μ*m.

We used immunofluorescence technique to manifest the macrophages (Figure 4). In Figure 4(a), it showed that the macrophages labeled with CD68 were located in the submucosa, myenteric, and muscular layers. The expression of CD68+ cells was almost the same in each group.

M1 macrophages were marked by CD86 (Figure 4(b)). There were few M1 macrophages distributed in the submucosa, myenteric, and muscular layers in the control group. In the DM group, a lot of CD86+ cells were observed in the stomach wall. At the same time, this situation still could be seen in the DM+SGEP group. Thus, only a very few CD86-positive cells could be found in the DM+GEP groups.

Co-expression of CD206 and HO-1 were exhibited the M2 macrophages in stomach wall (Figure 4(c)). There was an abundance of CD206+/HO-1+ cells in the control group. Quite a few CD206-positive and HO-1-positive cells were observed in the DM and DM+SGEP groups, but a dramatically increased number of CD206+/HO-1+ cells were shown in the DM+GEP groups.

### 3.3. Effects of GEP on Nrf2/HO-1 Pathway

**Figure 5:**
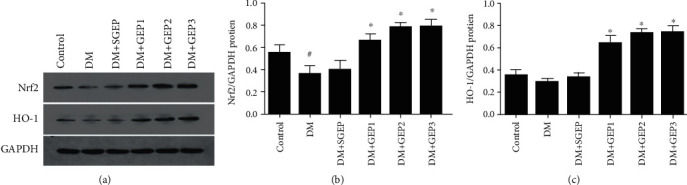
Expression of nuclear factor erythroid 2-related factor 2 (Nrf2) and hemeoxygenase-1 (HO-1) protein in the gastric antrum. (a) The expressions of Nrf2 and HO-1 were analyzed by western blot. (b, c) The representative quantification of Nrf2 and HO-1 protein was statistically evaluated (*n* = 6 per group). ^#^Compared with the control group, *P* < 0.05. ^∗^Compared with the DM group, *P* < 0.05.

As shown in Figure 5, the expression of Nrf2 protein in the DM group was significantly decreased compared with that in the control group (*P* = 0.020), but HO-1 was not changed (*P* = 0.365). Compared with the DM group, no significant difference in the expression of Nrf2 and HO-1 protein could be found in the DM+SGEP group (*P* = 0.618, *P* = 0.516). However, they were dramatically increased in the DM+GEP groups compared with the DM group (for Nrf2: *P* = 0.001, *P* < 0.001, and *P* < 0.001; for HO-1: all *P* < 0.001).

### 3.4. Effects of GEP on the Phosphorylation of NF-*κ*B

**Figure 6:**
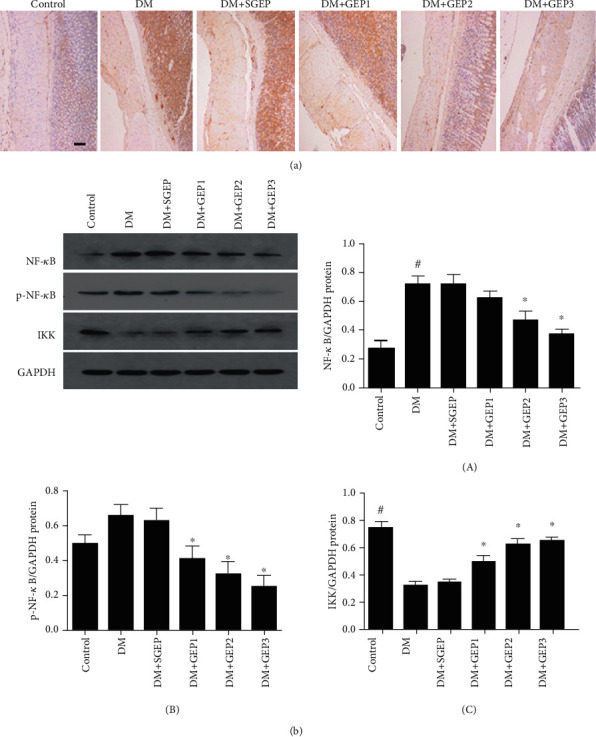
Effect of GEP on the phosphorylation of NF-*κ*B in the gastric antrum. (a) The NF-*κ*B expression was investigated by immunohistochemical staining. Scale bars = 500 *μ*m. (b) The expressions of NF-*κ*B and p-NF-*κ*B were analyzed (*n* = 6 per group). ^#^Compared with the control group, *P* < 0.05. ^∗^Compared with the DM group, *P* < 0.05.

Immunohistochemical technique was employed to evaluate NF-*κ*B expression (Figure 6(a)). There was a part of NF-*κ*B+ nucleus in each layer of the control group. In the DM and DM+SGEP groups, lots of NF-*κ*B+ nucleus and cytoplasm were demonstrated in the stomach wall. However, the expression of NF-*κ*B+ nucleus in the DM+GEP2 and DM+GEP3 groups was significantly reduced compared with that in the DM group.

To further reveal the phosphorylation of NF-*κ*B, western blot analysis was applied to detect the NF-*κ*B, p-NF-*κ*B, and IKK expression in the stomach wall (Figure 6(b)). Compared to the control group, the expression of NF-*κ*B was obviously upregulated in the DM group (*P* < 0.001). There were no significant differences for the expression of NF-*κ*B among the DM, DM+SGEP, and DM+GEP1 groups (*P* = 0.966, 0.180). Compared with that in the DM group, the protein expression level of NF-*κ*B in the DM+GEP2 and DM+GEP3 groups were evidently reduced (*P* = 0.002 and *P* < 0.001).

P-NF-*κ*B (p-p65) is the phosphorylation form of NF-*κ*B (p65). Although the expression of p-NF-*κ*B protein was increased in the DM group (*P* = 0.084) and the DM+SGEP group (*P* = 0.143), they still made no sense compared with the control group. Compared with the DM group, the p-NF-*κ*B protein expression had a significant difference in the DM+GEP group (*P* = 0.010, 0.001, and 0.000).

NF-*κ*B activation depends on the I*κ*B kinase (IKK) complex. In the DM group, the expression of IKK was dramatically downregulated compared with the control group (*P* < 0.001). However, there were no marked differences between the DM group and the DM+SGEP group (*P* = 0.703). Greatly, when compared with the DM group, the IKK expression was distinctly upregulated in the DM+GEP groups (*P* = 0.003, <0.001, and <0.001).

### 3.5. Effects of EA on the Level of MDA, SOD, and ROS

**Figure 7:**
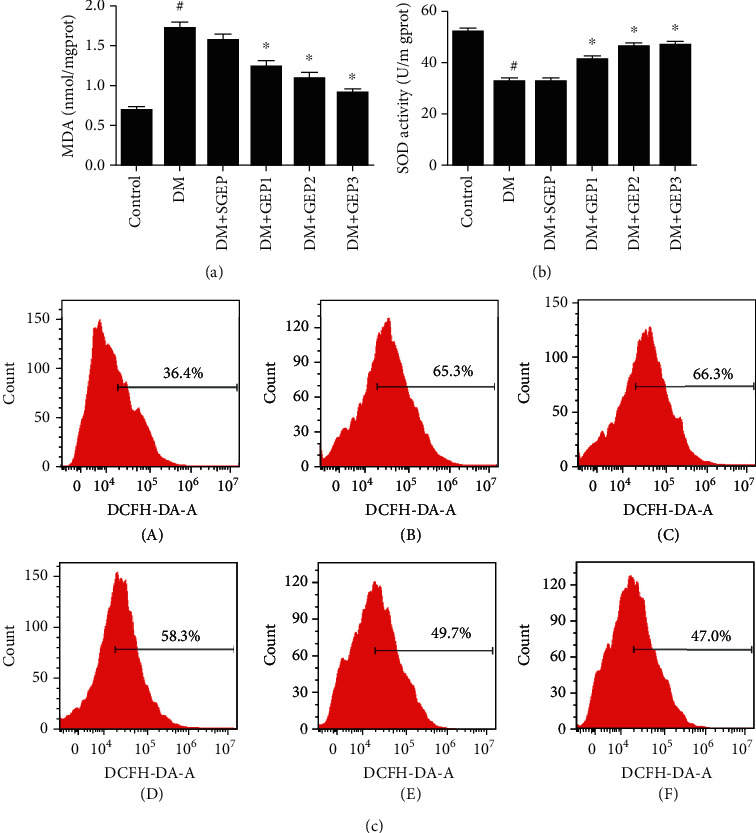
Measurement of malonaldehyde (MDA), superoxide dismutase (SOD), and reactive oxygen species (ROS) levels in different groups. (a) The MDA level was elevated in each group (*n* = 6 per group). ^#^Compared with the control group, *P* < 0.05. ^∗^Compared with the DM group, *P* < 0.05. (b) The SOD level was shown in each group (*n* = 6 per group). ^#^Compared with the control group, *P* < 0.05. ^∗^Compared with the DM group, *P* < 0.05. (c) The expression level of ROS from gastric antrum tissue in different groups was measured (*n* = 3 per group).

MDA and SOD were detected following the instruction of the manufacturer (Figures 7(a) and 7(b)). Compared with the control group, the MDA level was significantly elevated in the DM group (*P* < 0.001). There was no significant difference between the DM group and the DM+SGEP group (*P* = 0.18). However, the MDA level was dramatically reduced in the DM+GEP groups (all *P* < 0.001) compared with the DM group.

The SOD level in the stomach tissue was decreased distinctly in the DM group compared with the control group (*P* < 0.001). No obvious difference was found between the DM group and the DM+SGEP group (*P* = 0.888). However, the SOD level was clearly upregulated in the DM+GEP groups compared with the DM group (all *P* < 0.001).

The changes of ROS in the gastric antrum cells were detected by DCFH-DA fluorescence probe (Figure 7(c)). Compared with that in the control group, the expression of ROS in the DM group was significantly increased, the fluorescence signal of DCF was significantly enhanced, and the main peak of ROS shifted to the right side of the baseline. The same was seen in the DM+SGEP group. The fluorescence signal intensity in the DM+GEP groups was gradually weakened, and the main peak of ROS shifted to the left side of the baseline.

## 4. Discussion

In the present study, we demonstrated that GEP could decrease the apoptosis of ICCs via the improvement of phenotypic polarization of M2 macrophages, which promoted the activation of the Nrf2/HO-1 pathway and ameliorated the phosphorylation of NF-*κ*B. The effects of GEP were determined by frequency not pulse width of electrical stimulation. These underlying mechanisms indicated that GEP could reduce the oxidative stress, thus presenting a protective effect on ICCs.

Previous studies have demonstrated that loss and injury of ICCs have been verified in diabetic gastroparesis [2, 3, 20–22]. A further study reported that apoptotic ICC in the normal human colon was an ongoing process and the numbers of apoptotic ICC could be found [23]. The SCF/c-kit pathway is the basic guarantee to maintain of ICC quantity. We previously have reported that apoptosis of ICCs could be observed in the stomach and colon of diabetic rats, with downregulated SCF/c-kit pathway [6, 24, 25]. Wang et al. revealed that synchronized dual-pulse GES could decrease the apoptosis of enteric neurons in vagotomized rats [26]. The survival of ICCs could be rescued by long-pulse GES in light of our previous work [4, 5]. In the present study, we documented apoptosis of ICCs in the stomach of gastroparesis by immunofluorescence technique and GEP intervention could lessen the numbers of apoptotic of ICCs, with upregulated SCF/c-kit pathway. It implied that GEP played an antiapoptotic role of ICCs and further contributed to improved gastric motility.

To our knowledge, macrophages play a great role in gastric motility. Cipriani et al. found that diabetic *Csf1^op/op^* mice lacking macrophages were protected against the development of delayed gastric emptying [7]. A study also showed that delayed gastric emptying could be improved through phenotypic polarization of M2 macrophages [8]. Choi et al. revealed that CD206-positive M2 macrophages that expressed HO-1 protected against diabetic gastroparesis in mice [9]. Furthermore, Bernard et al. illustrated that low numbers of CD206-positive cells associated with loss of ICC in the gastric body of patients with diabetic gastroparesis [10]. Activation of macrophages with high levels of HO-1 expression protects against development of delayed gastric emptying in animal models of diabetes, while activation of macrophages that do not express HO-1 is linked to neuromuscular cell injury [11]. In our study, we observed different subtypes of macrophages and found that GEP increased the number of M2 macrophages and decreased the number of M1 macrophages, while the total number of macrophages was maintained. This result manifested that GEP promoted phenotypic polarization of M2 macrophages.

Oxidative stress plays a pivotal role in the development of diabetic complications, and antioxidant treatment could be a potential therapeutic procedure [27, 28]. Activation of the Nrf2/HO-1 signaling pathway contributes to the protective effects against oxidative stress-induced DNA damage and apoptosis [29]. As a major protective mechanism against oxidative stress, HO-1 has been found to protect ICCs and prevent against gastroparesis. Choi et al. reported that HO-1 did not upregulate in the diabetic mice that developed delayed gastric emptying and loss of HO-1 upregulation increased levels of reactive oxygen species. Induction of HO-1 protected ICCs from oxidative stress and reversed diabetic gastroparesis in NOD mice [13]. In a rat model of streptozotocin-induced diabetes, impaired HO-1 induction in the gastric antrum induced disruption of the ICC network; thus, upregulated HO-1 expression could significantly restore the previously reduced ICC in the gastric antrum [14]. Although it is reported that EA could improve the expression of HO-1 in diabetic gastroparesis rats [15], the effects of GEP were to investigate as these two interventions had different mechanisms. We clarified that different pulse width with similar frequency GEP could promote the expression of the Nrf2/HO-1 signaling pathway, indicating the possible mechanism that GEP restore ICC in diabetic gastroparesis rats.

The NF-*κ*B signaling pathway was considered both anti- and prooxidant roles in the setting of oxidative stress [30]. The NF-*κ*B pathway was activated in diabetic gastroparesis, and oxidative stress was produced, resulting in the decreased expression of SCF/c-kit and the increase of ICC apoptosis [17]. Simultaneously, HO-1 could inhibit the phosphorylation of NF-*κ*B p65, promote the binding of anti-apoptotic genes to NF-*κ*B, and promote gene transcription to play an antiapoptotic role [19]. Our results showed that the expression of NF-*κ*B and phosphorylated NF-*κ*B p65 was increased in diabetic rats, with the expression of IKK (a central regulator of NF-*κ*B activation) which was decreased. However, GEP could significantly suppress the phosphorylation of NF-*κ*B p65, indicating that HO-1 may play an antiapoptotic role by inhibiting the phosphorylation of NF-*κ*B p65.

Oxidative stress, an imbalance between the production of reactive oxygen species (ROS) and antioxidant defenses, is considered seriously in the production of tissue damage in diabetes mellitus [31]. MDA has been taken as an indirect marker of oxidative stress [32]. In our experiments, MDA was significantly increased in diabetic rats, which was consistent with previously reported findings [15], and GEP could lower the level of MDA. SOD, a main antioxidant enzyme, plays a role in the destruction of free superoxide radicals and blocks free radical-induced damage [33]. The SOD activity was significantly decreased in diabetic rat, while GEP could improve the activity of SOD. The present study indicates that GEP could ameliorate the balance of ROS production and antioxidant defenses. ROS are free radicals of unstable molecule that contains oxygen which may cause damage and cell death [34]. A previous study showed that oxidative stress resulted from high blood glucose through abundant ROS generation, which caused the complications of diabetes [35, 36]. In additional, we detected the ROS by flow cytometry and found that GEP could decrease the expression of ROS, suggesting that apoptosis of ICCs was reduced by GEP on the basis of lowering the ROS. This point coincides with the previous study [37].

## 5. Conclusions

Apoptosis of ICCs was verified in a diabetic rat with disturbance of oxidative stress, which were associated with the decreased number of M2 macrophages and downregulated Nrf2/HO-1 pathway. The progress was facilitated by activated NF-*κ*B pathway resulting ICC apoptosis. However, GEP with different pulse width but the same frequency could reduce apoptosis of ICCs and promote phenotypic polarization of M2 macrophages via antioxidative stress effects in diabetic rats, which also optimized the Nrf2/HO-1 and NF-*κ*B pathway. These results suggest that the protective effects of GEP targeting antioxidative stress might be a potential therapeutic strategy for diabetes dysmotility.

## Data Availability

The data used to support the study can be available upon request.

## Abbreviations

GEP: Gastric electrical pacing
ICCs: Interstitial cells of Cajal
HO-1: Heme oxygenase-1
DM group: Diabetic group
DM+SGEP group: Diabetic with sham GEP group
DM+GEP1 group: Diabetic with GEP1 group
DM+GEP2 group: Diabetic with GEP2 group
DM+GEP3 group: Diabetic with GEP3 group
EA: Electroacupuncture
GES: Gastric electrical stimulation
SCF: Stem cell factor
Nrf2: Nuclear factor erythroid 2-related factor 2
MDA: Malonaldehyde
ROS: Reactive oxygen species
SOD: Superoxide dismutase.
